# Early sepsis markers in patients admitted to intensive care unit with moderate-to-severe diabetic ketoacidosis

**DOI:** 10.1186/s13613-020-00676-6

**Published:** 2020-05-19

**Authors:** Florian Blanchard, Judith Charbit, Guillaume Van der Meersch, Benjamin Popoff, Adrien Picod, Regis Cohen, Frank Chemouni, Stephane Gaudry, Helene Bihan, Yves Cohen

**Affiliations:** 1 Medical-Surgical Intensive Care Unit, Avicenne University Hospital, AP-HP, Paris 13 University, Sorbonne Paris Cité, 125 rue Stalingrad, 93000 Bobigny, France; 2 Department of Endocrinology, Diabetology, Metabolic Disease, Avicenne University Hospital, AP-HP, Paris 13 University, Sorbonne Paris Cité, CRNH-IdF, 125 rue Stalingrad, Bobigny, France; 3 Department of Anesthesiology and Critical Care, Rouen University Hospital, Rouen, France; 4 Department of Endocrinology, Delafontaine Hospital, 2 rue du Dr Delafontaine, Saint-Denis, France; 5 Gustave Roussy, Médecine Intensive Réanimation, 94805 Villejuif, France; 6 Sorbonne University, INSERM, Remodeling and Repair of Renal Tissue, UMR S1155, Tenon Hospital, Paris, France

**Keywords:** Diabetic ketoacidosis, Sepsis, Bacterial infection, Procalcitonin, Inflammation, Biomarkers

## Abstract

**Background:**

Bacterial infections are frequent triggers for diabetic ketoacidosis. In this context, delayed antibiotic treatment is associated with increased morbidity and mortality. Unnecessary administration of antimicrobial therapy might however, also negatively impact the prognosis. The usefulness of sepsis markers in diabetic ketoacidosis has not been assessed. Thus, we sought to investigate diagnostic performances of clinical and biological sepsis markers during diabetic ketoacidosis.

**Methods:**

In this monocentric retrospective cohort study, all consecutive episodes of diabetic ketoacidosis (defined as pH ≤ 7.25, glycaemia > 300 mg/dL and presence of ketones) admitted in intensive care unit were included. A proven bacterial infection was defined as bacteriological documentation on any bacterial sample. Clinical (presence of fever: temperature > 38 °C and presence of hypothermia: temperature < 36 °C) and biological markers (whole blood count, neutrophils count, neutrophils-to-lymphocytes count ratio and procalcitonin), recorded at admission, were compared according to the presence or absence of a proven bacterial infection.

**Results:**

Between 2011 and 2018, among 134 episodes of diabetic ketoacidosis, 102 were included (91 patients). Twenty out of 102 were infected. At admission, procalcitonin (median: 3.58 ng/mL vs 0.52 ng/mL, *p* < 0.001) and presence of fever (25% vs 4%, *p* = 0.007) were different between episodes with and without proven bacterial infection in both univariate and multivariate analysis. Whole blood count, neutrophils count, neutrophils-to-lymphocytes count ratio and presence of hypothermia were not different between both groups. The diagnostic performance analysis for procalcitonin revealed an area under the curve of 0.87 with an optimal cutoff of 1.44 ng/mL leading to a sensitivity of 0.90 and a specificity of 0.76. Combining procalcitonin and presence of fever allowed to distinguish proven bacterial infection episodes from those without proven bacterial infection. Indeed, all patients with procalcitonin level of more than 1.44 ng/mL and fever had proven bacterial infection episodes. The presence of one of these 2 markers was associated with 46% of proven bacterial infection episodes. No afebrile patient with procalcitonin level less than 1.44 ng/mL had a proven bacterial infection.

**Conclusion:**

At admission, combining procalcitonin and presence of fever may be of value to distinguish ketoacidosis patients with and without proven bacterial infection, admitted in intensive care unit.

## Introduction

Diabetic ketoacidosis (DKA) accounts for 4–9% of all hospital discharge summaries among diabetic patients [1, 2]. Despite a 50% drop in mortality since 1980 due to standardized protocols [3], recent studies still report a mortality rate of about 2–5%, mostly depending on the age of the patients [4, 5]. Therapeutic guidelines on DKA almost only focus on insulin administration and hydroelectrolytic supplementation [6, 7].

Discontinuation of insulin therapy and infections are the most frequent triggering factors [2, 5, 8]. Bacterial infections—urinary tract infections in the first place, followed by pneumonia—explain up to 50% of ketoacidosis cases [6, 8, 9]. In the context of DKA, bacterial infections are reported to increase both mortality [10] and length of stay [9]. Then, early detection of bacterial infections associated with adequate antibiotic treatments are key elements to improve patient outcomes. DKA itself can however mimic infections [10] and differentiation of septic from non-septic inflammatory response may be difficult. Clinical suspicion of infection can hardly be used and many patients are over-treated with antibiotics leading to inadequate treatment costs, side effects and bacteriological resistance. The development of bacteriological resistance is all the more worrying for these patients admitted for DKA knowing that 20 to 40% of them will develop a new episode of infection in the future [3, 5, 11].

To our knowledge, no study has already assessed the usefulness of sepsis markers in DKA. The constitutive signs of the systemic inflammatory response syndrome are considered to have a poor specificity [12]. Tachycardia and polypnea can be easily integrated in DKA pathophysiology. Hypothermia, fever, and white blood cell count abnormalities are usually considered when assessing septic status. However, none of those signs ever were found to be relevant to distinguish infected from non-infected patients during DKA. Procalcitonin (PCT) is actually one of the major relevant markers for the diagnosis of bacterial infections. PCT is daily used for antibiotic decisions in patients with respiratory tract infections and sepsis [13–15]. Nevertheless, some preliminary data suggest that compared with non-diabetics, PCT positive threshold could be higher in diabetic patients, especially during hyperglycemic crisis [16, 17]. We therefore conducted a retrospective study in which we sought to investigate the diagnostic performance of different sepsis markers (including PCT) to predict bacterial infection in the first 2 days of admission in intensive care unit (ICU) for DKA.

## Materials and methods

### Design and patients

This was a retrospective study performed in the ICU of Avicenne hospital, a French tertiary hospital, in the Paris area. All consecutive patients hospitalized for moderate-to-severe DKA between January 2011 and March 2018 were included. DKA was defined as a glucose concentration > 300 mg/dL (16.7 mmoL/L) (either serum or capillary), a pH ≤ 7.25 or a serum bicarbonate concentration < 15 mmol/L, and the presence of ketones [acetoacetate] (either in the blood or the urine) [11, 18]. Patients were not included if they had one or more criteria known to increase PCT without any indication of bacterial infection (medullary thyroid carcinoma, small cell lung cancer, cardiac arrest, heat stroke, pancreatitis, malaria, notion of fungal infection, severe trauma) [19].

### Ethics

The study was approved by the “Comité d’éthique pour la recherche en Anesthésie-Réanimation” in Paris, France (reference: IRB 00010254 - 2018 – 029). As a retrospective study of routinely collected and anonymized data, consent was not required, and patients were only informed by letter of their enrollment in the studies.

### Data collection

From charts, we extracted the following data at admission (D0): clinical parameters such as history and type of diabetes, comorbidities, diabetes complications, medication, temperature, and biological data such as pH, bicarbonate, first glycemia available (either by venous or capillary blood punctures), ketonemia, sepsis markers (see below). A follow-up of those parameters was collected on day 2 (D2) when available. Triggering factors of DKA, length of stay in ICU and hospital, and hospital mortality were also collected. Clinical and biological sepsis markers were assessed on D0 and D2. As part of the systemic inflammatory response syndrome, temperature and white blood cell count were collected. Temperature was measured in the armpit area with the addition of a correcting factor (+ 0.5 °C) [20]. Temperature was then classified: fever (> 38 °C); apyrexia (36–38 °C) and hypothermia (< 36 °C) [21]. From the whole blood count, white blood cell count (WBC), neutrophil blood count and neutrophils-to-lymphocytes count ratio (NLCR) were extracted. Patients were considered to have leukocyte abnormalities in case of WBC > 12.0 G/L or < 4.0 G/L [21]. Plasma PCT concentrations were also determined at D0 and D2. Plasma PCT concentrations were measured using an automated immunofluorescent assay (PCT KRYPTOR^®^, ThermoFisher scientific). PCT concentration was considered normal if below 0.5 ng/mL. C-reactive protein was not collected due to the high number of missing data.

### Definition of “Proven bacterial infection”

Patients with a bacteriological documentation on any bacterial sample (urine culture, sputum analysis, blood culture and other specific sample cultures) were classified as having a “proven bacterial infection” (PBI). Bacterial samples were only requested in case of suspected infection.

### Statistical analysis

A descriptive analysis was performed for all episodes and according to infection status: “with PBI” and “without PBI”. We performed a univariate analysis to compare these 2 subgroups of patients. Quantitative variables are expressed as median [interquartile range (IQR), 25–75%] and compared using the Mann–Whitney *U* test. Categorical variables are expressed as numbers (%) and compared using the Fisher’s exact test. We performed a multivariate logistic regression analysis to assess the relationship between sepsis markers and infection status. Interactions between significant variables during the univariate analysis (threshold of *p* < 0.05) were tested. As sepsis markers on D0 and D2 are coupled, we used 2 different models integrating the significant variables at each time point (D0 and D2). The multivariate analysis was done on a complete case analysis. A sensitivity analysis with a multiple imputation was achieved to deal with missing data. Receiver operating characteristics (ROC) curves analysis was performed to assess the ability of sepsis markers to predict infection. Optimal cutoff values were chosen to maximize sensitivity and specificity using the Youden index. A *p* value of ≤ 0.05 was considered to be statistically significant. Statistical analyses were carried out using R version 3.4.1 for Windows^®^ (https://www.r-project.org, accessed June 2019).

## Results

### Patients

**Fig. 1:**
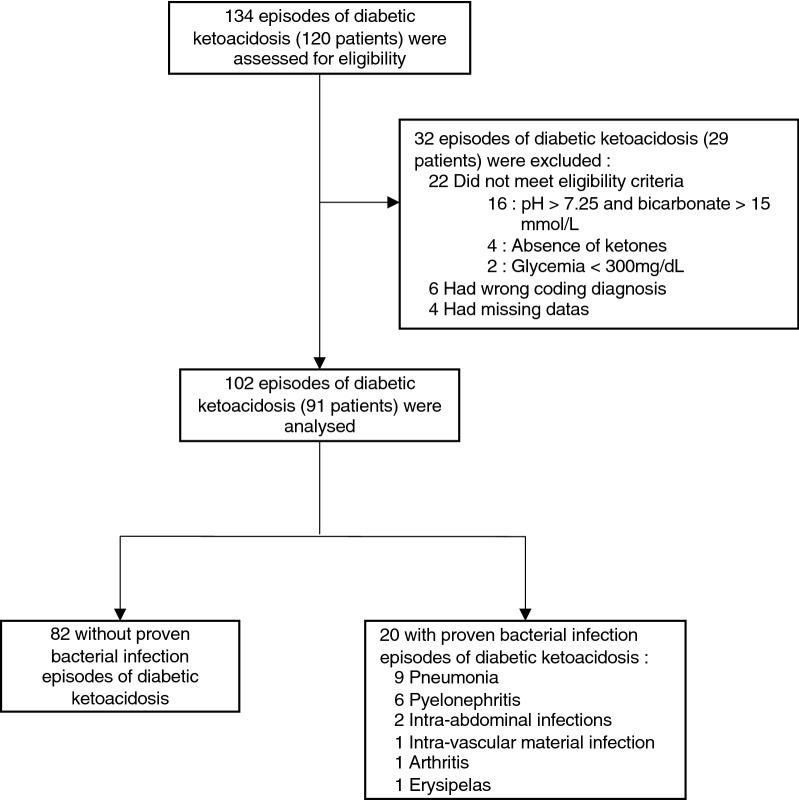
Flowchart of study population and sample size. Shown is the recruitment of the cohort

Between January 2011 and March 2018, 134 episodes of DKA (120 patients) were admitted in the ICU. We did not include 32 episodes (29 patients) because of the absence of eligibility criteria, wrong coding diagnosis or missing data (Fig. 1). The remaining 102 episodes (91 patients) were included in the analysis. There were 50 (49%) males and 52 (51%) females, with a mean age of 46 years (29–58 years) (Table 1). Type 1 diabetes was the most frequent type of diabetes (*n* = 60 episodes, 59%) and inaugural DKA accounted for 18% (*n* = 18 episodes). Eight patients (9%) had recurrent episodes of DKA (2 episodes: 6 patients, 3 episodes: 1 patient, 4 episodes: 1 patient). Triggering factors were poor compliance to antidiabetic treatments and bacterial infection, for 50% and 20% of the episodes, respectively. For 21 episodes (21%), no triggering factor was identified. At ICU admission, median pH and bicarbonate were 7.14 [7.05–7.24] and 6.0 mmol/L [3.5–10.4 mmol/L], respectively. On D2, ketoacidosis was corrected as shown by a median pH of 7.41 [7.38–7.43] and glycemia 6.6 mmol/L [5.2–11.0 mmol/L].

**Table 1:** Demographic and clinical baseline characteristics of the episodes

Variables	All cohort (*n* = 102)	Episodes with PBI (*n* = 20)	Episodes without PBI (*n* = 82)	*p* value^a^
Age, year, median [IQR]	47 [29–58]	56 [48–64]	41 [28–57]	*0.003*
Males, *n* (%)	50 (49%)	9 (45%)	41 (50%)	
Body mass index, kg/m^2^, median [IQR]	23.65 [20.97–26.54]	24.49 [20.89–30.25]	23.63 [21.05–26.30]	
Inaugural diabetes ketoacidosis, *n* (%)	18 (18%)	7 (35%)	11 (13%)	*0.045*
Type 1 diabetes mellitus, *n* (%)	61 (60%)	7 (35%)	54 (66%)	*0.021*
Type 2 diabetes mellitus, *n* (%)	23 (23%)	6 (30%)	17 (21%)	ns
Insulin-dependent diabetes mellitus, *n* (%)	72 (71%)	9 (45%)	63 (77%)	*0.006*
Comorbidities				
Hypertension, *n* (%)	30 (29%)	5 (25%)	25 (31%)	
Dyslipidemia, *n* (%)	19 (19%)	4 (20%)	15 (18%)	
Ischemic heart disease, *n* (%)	7 (7%)	2 (10%)	5 (6%)	
Diabetic retinopathy, *n* (%)	27 (27%)	4 (20%)	23 (28%)	
Chronic kidney disease, *n* (%)	24 (24%)	5 (25%)	19 (24%)	
Smoking, *n* (%)	43 (42%)	5 (25%)	38 (46%)	ns
Alcohol, *n* (%)	24 (24%)	4 (20%)	20 (24%)	
Medications				
Insulin, *n* (%)	72 (71%)	9 (45%)	63 (77%)	*0.006*
Metformin, *n* (%)	23 (23%)	6 (30%)	17 (21%)	ns
Sulfonylurea, *n* (%)	8 (8%)	3 (15%)	5 (6%)	ns
No antidiabetic, *n* (%)	18 (18%)	7 (35%)	11 (13%)	*0.045*
Triggering factors				
Poor compliance to antidiabetic treatment, *n* (%)	51 (50%)			
No triggering factors, *n* (%)	21 (21%)			
Infection, *n* (%)	20 (20%)			
Others, *n* (%)	11 (11%)			
Clinical and biological data				
pH, median [IQR]	7.14 [7.05–7.24]	7.15 [7.02–7.27]	7.14 [7.05–7.22]	ns
Bicarbonate, mmol/L, median [IQR]	6.00 [3.50–10.40]	8.00 [4.15–10.55]	5.90 [3.30–10.30]	ns
Glycemia, mmol/L, median [IQR]	27.5 [26.1–30.6]	27.5 [25.1–29.0]	27.5 [26.1–30.9]	
Ketonemia, mmol/L, median [IQR]	6.0 [5.1–6.9]	5.00 [3.95–5.93]	6.1 [5.3–7.0]	*0.007*
SAPS II, median [IQR]	29 [21–40]	45 [35–58]	26 [20–36]	*< 0.001*
Treatments and outcomes				
Antibiotics treatments, *n* (%)	45 (44%)	20 (100%)	25 (31%)	*< 0.001*
ICU length of stay, day, median [IQR]	2 [1–4]	7 [6–12]	2 [1–3]	*< 0.001*
Hospital length of stay, day, median [IQR]	9 [6–14]	20 [12–24]	8 [6–12]	*< 0.001*
Death, *n* (%)	2 (2%)	1 (5%)	1 (1%)	

*IQR* interquartile range 25–75%, *ICU* intensive care unit, *PBI* proven bacterial infection, *SAPS II* Simplified Acute Physiology Score II

^a^Significant difference (*p* < 0.05) between episodes with and without proven bacterial infection are reported in the “*p* value” column

### Infections

Among the 102 (20%) episodes, 20 were classified PBI. These patients were older and had more frequently inaugural DKA compared with patients without PBI (Table 1). On D0, ketonemia was significantly lower and the severity score [simplified acute physiology score II (SAPS II)] was significantly higher for PBI episodes. On D2, correction of DKA was similar in both groups. Antibiotics were administered to 45 episodes: 20/20 (100%) for PBI episodes versus 25/82 (31%) for episodes without PBI. Length of stay in ICU and hospital were higher for PBI episodes (7 vs 2 days, *p* < 0.001 and 20 vs 8 days, *p* < 0.001, respectively).

### Sepsis markers at ICU admission

#### Univariate analysis

On D0, temperature, fever rate and PCT level were significantly higher in PBI episodes compared with episodes without PBI (36.9 [36.2–38.0] vs 36.4 [35.7–36.8] °C; 5 (25%) vs 3 (4%) episodes and 3.58 [1.87–11.24] vs 0.52 [0.19–1.38] ng/mL, respectively). The other sepsis markers (hypothermia, WBC, neutrophil count, leukocytes abnormalities and NLCR) did not significantly differ between groups (Table 2).

**Table 2:** Association between sepsis markers and presence of proven bacterial infection at admission and day 2

Variables	Admission			Day 2		
	Episodes with PBI (*n* = 20)	Episodes without PBI (*n* = 82)	*p*-value^a^	Episodes with PBI (*n* = 20)	Episodes without PBI (*n* = 82)	*p*-value^a^
Temperature, °C, median [IQR]	36.9 [36.2–38.0]	36.4 [35.7–36.8]	*0.032*	38.4 [37.1–39.0]	37.0 [36.8–37.3]	*<0.001*
Fever^b^, *n* (%)	5 (25%)	3 (4%)	*0.007*	12 (60%)	7 (9%)	*<0.001*
Hypothermia^c^, *n* (%)	4 (20%)	26 (32%)	0.410	0 (0%)	1 (1%)	1
WBC, G/L, median [IQR]	16.85 [14.25–22.15]	15.40 [12.30–22.50]	0.606	13.05 [8.68–18.23]	8.15 [6.68–10.20]	*<0.001*
Leukocyte abnormalities^d^, n (%)	18 (90%)	62 (76%)	0.232	11 (55%)	14 (17%)	*0.001*
Neutrophils count, G/L, median [IQR]	13.30 [12.01–18.24]	13.71 [9.69–20.88]	0.673	10.79 [7.39–16.64]	5.38 [3.60–7.62]	*<0.001*
NLCR; median [IQR]	14.04 [8.79–19.07]	11.40 [5.78–19.27]	0.359	11.54 [7.63–23.99]	2.84 [1.56–4.96]	*<0.001*
Procalcitonin, ng/mL, median [IQR]	3.58 [1.87–11.24]	0.52 [0.19–1.38]	*<0.001*	7.43 [2.63–22.70]	0.42 [0.14–1.42]	*<0.001*

*PBI* proven bacterial infection, *IQR* interquartile range 25–75%, *WBC* white blood cell count, *NLCR* neutrophils-to-lymphocytes count ratio

^a^Significant difference (*p* < 0.05) between episodes with and without proven bacterial infection are reported in the “*p*-value” column

^b^Fever: temperature > 38 °C

^c^Hypothermia: temperature < 36 °C

^d^Leukocyte abnormalities: white blood cell count > 12,000/mm^3^ or < 4000/mm^3^

#### Multivariate analysis

Adjustment for age, type of diabetes, insulin treatment, ketonemia and SAPS II (significant variables during the univariate analysis) revealed that PCT [OR = 1.27, 95% confidence interval (IC95) [1.04–1.63] (*p* = 0.029) for each point of increase of PCT] and presence of fever [OR = 27.86, IC95 [1.97–887.92] (*p* = 0.023)] were independently associated with PBI. Ketonemia was unavailable for 16 episodes (4 episodes with PBI (20%) vs 12 episodes without PBI (15%), *p* = 0.512). Those 16 episodes were included in the study on the basis of the presence of ketones in urine. A sensitivity analysis with multiple imputation for missing ketonemia data confirmed the previous multivariate analysis.

#### ROC curves

**Fig. 2:**
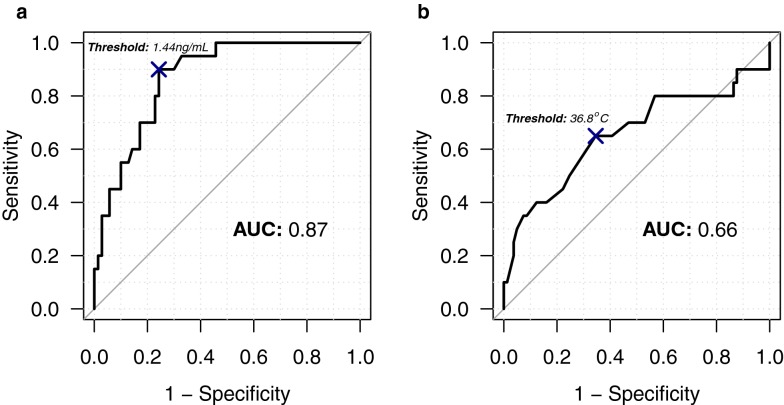
Receiver operating characteristics curve of procalcitonin (PCT) (**a**) and temperature (**b**) at admission. For PCT, the area under curve (AUC) was 0.87 with optimal cutoff at 1.44 ng/mL leading to a sensitivity and specificity of 0.90 (IC95 [0.75–1.00]) and 0.76 (IC95 [0.66–0.84]), respectively. Positive and negative likelihood ratios were 3.75 and 0.13, respectively. For temperature, the area under curve (AUC) was 0.66 with optimal cutoff at 36.8 °C leading to a sensitivity and specificity of 0.65 (IC95 [0.45–0.85]) and 0.65 (IC95 [0.56–0.75]), respectively. Positive and negative likelihood ratios were 1.86 and 0.54, respectively

The area under the curve (AUC) for PCT was 0.87 (IC95 [0.79–0.94]) (Fig. 2a). The optimal threshold was obtained at 1.44 ng/mL leading to a sensitivity (Se) of 0.90 (IC95 [0.75–1.00]) and specificity (Sp) of 0.76 (IC95 [0.66–0.84]). The AUC for temperature was 0.66 (IC95 [0.50–0.81]) with an optimal threshold of 36.8 °C (Se: 0.65 IC95 [0.45–0.85]; Sp: 0.65 IC95 [0.56–0.75]) (Fig. 2b).

#### Performance of PCT and fever (> 38.0 °C) for the diagnosis of infection (Fig. 3)

Association of a high PCT level (PCT > 1.44 ng/mL) with or without presence of fever for the diagnosis of PBI was then investigated. All episodes with a PCT level of more than 1.44 ng/mL (defined using ROC curves, see above) and fever were PBI episodes. The presence of one of these 2 markers was associated with 46% of PBI episodes. No afebrile patient with PCT level less than 1.44 ng/mL had a PBI.

**Fig. 3:**
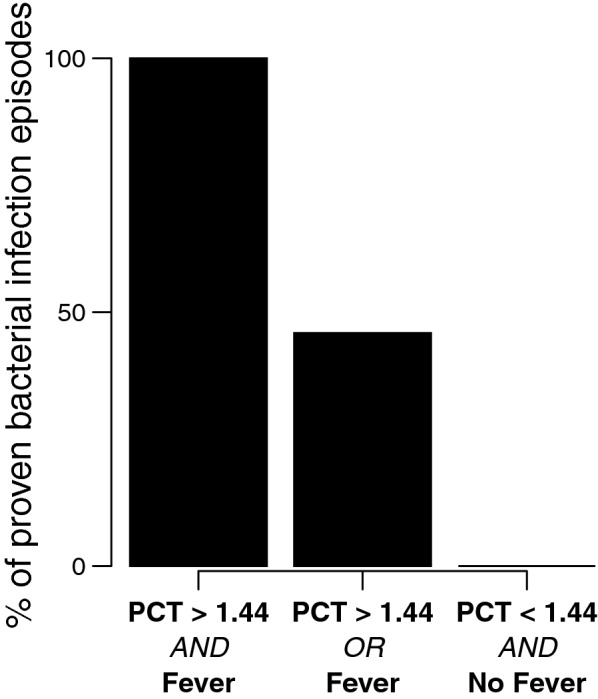
Procalcitonin (PCT) and fever as markers of proven bacterial infection at admission. Results are shown in percent of episode with proven bacterial infection over total episode presenting both (100%), either one (46%) or none (0%) of PCT >1.44 ng/mL and fever. PCT is expressed in ng/mL. Fever is retained in case of temperature > 38 °C

### Sepsis markers at D2

#### Univariate analysis

On D2, more differences appeared between the two groups. Indeed, temperature, fever rate, WBC, neutrophil count, leukocytes abnormalities, NLCR and PCT were significantly higher in PBI episodes (Table 2). Both groups were also different when looking at any episode of fever during the first 2 days of hospitalization (PBI episodes: 60% vs episodes without PBI: 9%, OR = 14.3 IC95 [4.0–58.0], *p* < 0.001). In PBI episodes, between D0 and D2, NLCR and PCT did not change significantly (*p* = 0.968 and *p* = 0.283, respectively), whereas in episodes without PBI, NLCR (D0: 13.42 vs D2: 5.37, *p* < 0.001) and PCT (D0: 0.51 ng/mL vs D2: 0.39 ng/mL, *p* = 0.005) significantly decreased (see Additional file 1: Tables S1 and S2).

#### Multivariate analysis

Adjustment for age, type of diabetes, insulin treatment and ketonemia and SAPS II during the multivariate analysis revealed that only PCT was independently associated with PBI episodes [OR = 4.45, IC95 [1.73–39.53] (*p* = 0.030) for each point of increase of PCT], whereas presence of fever and WBC were not.

#### ROC curves

The AUC for PCT was 0.91 (IC95 [0.84–0.99] with an optimal threshold at 2.78 ng/mL leading to a Se of 0.74 (IC95 [0.53–0.89] and a Sp of 0.96 (IC95 [0.89–1.00]) (see Additional file 1: Figure S1). The AUC for temperature, WBC, neutrophil count and NLCR were 0.79, 0.78, 0.84 and 0.87, respectively (see Additional file 1: Figure S2).

## Discussion

This is the first study to assess the diagnostic performance of different sepsis markers to predict proven bacterial infection for patients with DKA, admitted in ICU. Fever and high PCT (threshold above 1.44 ng/mL at D0) at ICU admission may help to identify patients with proven bacterial infection in the context of DKA.

The only clinical marker was temperature. Presence of fever on D0 and D2 was higher in PBI episodes as reported in previous studies [10]. Nevertheless, in episodes without PBI, body temperature ranged from 32.9 °C till 38.7 °C on D0. This huge variation may be explained by thermoregulatory function impairment in diabetic patients [22]. Hypothermia (temperature < 36 °C) was equally presented in both groups. In 1978 Gale et al. [23] reported 20 patients with hypothermia during DKA and observed a high mortality rate (60%). Hypothermia was also associated with infection [24]. In our study, we neither found an increase in mortality nor an association with sepsis for hypothermic patients.

Other classical sepsis markers were also found to be inefficient in our study to differentiate PBI episodes from those without PBI. We found a high WBC level on D0 (mostly composed of neutrophil polynuclears) in episodes without PBI. Such leukocytosis, as high as 57.0 G/L, has already been reported in several case reports [25, 26]. This result leads to reconsider the usefulness of WBC to predict bacterial infection at admission. Recently, the NLCR was proposed to be a more useful diagnostic tool than other blood tests to identify patients with bacterial infection [27]. However, in our study we did not highlight any difference for this marker between both groups at admission.

PCT, a precursor of calcitonin, is generated as part of the systemic response to bacterial infections [28]. Our study emphasized the relevance of PCT to predict infection, with a good predictive value above the level of 1.44 ng/mL at D0. In febrile patients admitted in the emergency department, Hausfater et al. [29] stated that a 0.2 ng/mL cutoff value for PCT had a low Se and Sp to diagnose bacterial infections (0.77 and 0.59). Wacker et al. [30] in their meta-analysis focusing on the accuracy and clinical value of PCT for diagnosis of sepsis in critically ill patients reported a Se and Sp of 0.77 and 0.79, respectively. Sager et al. [31] recently summarize the use of PCT to guide sepsis diagnosis. For critically ill patients, bacterial infection was considered to be “likely” when PCT level was 0.5–1.0 ng/mL and to be “very likely” above 1.0 ng/mL. In our study, PCT was accurate at the admission to distinguish PBI episodes from those without PBI with a sensitivity of 0.90 and a specificity of 0.76. However, the positive threshold seems to be higher than usual in our study (optimal cutoff on D0: 1.44 ng/mL). Previous studies already reported huge cutoff heterogeneity. For example, Wacker et al. [30] in their meta-analysis reported a median cutoff of 1.1 ng/mL (IQR 0.5–2.0 ng/mL). However, a participation of the hyperglycemic crisis in the increase of the PCT could not be excluded. Indeed, Aksu et al. [32] had reported a decrease in PCT level following a normalization of glycemia in patients with acute hyperglycemic crisis. In our study, we found an elevated a high level of PCT in patients without any proven bacterial infection, with a PCT drop following normalization of glycemia. High PCT levels were recently reported in different case reports or case series focusing on diabetes ketoacidosis without infection [33, 34]. A case series of 5 patients hospitalized for diabetes ketoacidosis reported PCT levels ranging from 6.87 to 30.47 ng/mL. Interestingly, this observation was not found in case of hyperosmolar hyperglycemic syndrome. This led the author to conclude that the augmentation of PCT in acute glycemic crisis may only be found during diabetes ketoacidosis [34].

In early management of DKA, traditional clinical (hypothermia) and biological (WBC, NLCR) signs of bacterial infection proved to be ineffective, probably because of the reported correlation between hyperglycemia crisis and inflammatory response. In non-diabetic patients, induced hyperglycemia led to an amplification of interleukin-6 (IL-6) and other pro-inflammatory markers [35]. Adding low doses of insulin avoids these alterations even with persistent hyperglycemia [35]. Compared with healthy controls, an induced hyperglycemia in diabetic patients results in a more pronounced secretion of pro-inflammatory cytokines such as tumor necrosis factor-α (TNF-α) and IL-6 [36]. Apart from any bacterial infection, TNF-α is known to induce the release of large amount of PCT in both animals [37] and humans [38]. These data may explain the increase of both PCT and WBC in episodes without PBI on D0. Combining PCT and presence of fever may help to be more specific. Indeed, only PBI episodes presented both signs, whereas there was no PBI episode with the absence of both signs (Fig. 3). On D2, after administration of insulin and correction of glycemia, the near normalization of PCT and WBC in episodes without PBI may be explained by the correction of this inflammatory state, allowing to distinguish two different patterns: episodes with and without PBI. In the former group, episodes of fever occur and high levels of PCT, WBC, neutrophil count and NLCR still persist on D2. In the latter, there is a decrease, if not a normalization, of all the aforementioned markers following the correction of glycemia. Thus, on D0, infection status could be based on PCT level and presence of fever regardless of WBC or hypothermia occurrence. On D2, after normalization of glycemia, usual markers recover their discriminating potential and can enable the reassessment of antibiotic prescriptions if started on D0.

This study has some limitations. First, it was a monocentric retrospective analysis which limits the generalizability of the results. However, PCT and WBC measurement were systematically assessed for every patient admitted for DKA in our center. Second, the sample size is small with a significant number of excluded patients (32 out of 134) for different issues (as reported in Fig. 1) that may have may induced bias. Third, we only studied episodes of proven bacterial infection defined by a positive bacterial culture. This definition of bacterial infection may have induced bias, since cultures may not be realized or false-negative. Indeed, 31% of non-PBI episodes received antibiotics. Although this may point to overtreatment for some cases, it may also point to a non-proven, but still present bacterial infection. Thus, some episodes classified as without PBI may not be truly free from bacterial infection. Fourth, C-reactive protein was not available to almost all of the patients in our cohort. C-reactive protein was already reported to be associated with infection in patients with severe DKA [10]. However, as we did with PCT, some authors reported an elevation of C-reactive protein in patients free of infection during DKA episodes [39]. In our institution, C-reactive protein is not measured due to its contradictory result for critical care patients [40]. Even if C-reactive protein would have been informative, we were not able to include it in our study. Finally, temperature was not measured centrally. Central temperature device (urinary catheter, esophageal temperature probe or Swan–Ganz catheter) are not used in the context of DKA due to their invasive nature. In our institution, when central temperature is not available, axillary temperature is measured. The mean difference between axillary temperature and pulmonary artery temperature is known to be 0.5 °C (− 0.4 to 1.2 °C) [41]. In order to estimate core temperature, we choose to add a correction factor of 0.5 °C to the axillary measured temperature [20]. Despite these limitations, our study was a “real-life” observation of the population and was consistent with previous studies regarding triggering factors [2], survival rate [4] and increased length of stay in infected patients [9]. Prospective studies will be needed to confirm the interest and the diagnostic thresholds of these markers. Thus, a prospective clinical randomized control trial incorporating a decision rule based on procalcitonin and fever to guide the prescription of antibiotics during DKA could be conducted to confirm the clinical added value of such attitude.

## Conclusions

At admission, our study showed that WBC, neutrophils count and hypothermia should not be taken into account in the diagnosis process of infection in diabetes ketoacidosis patients admitted in intensive care unit. Only fever and PCT (with a higher threshold than usual: 1.44 ng/mL) may help distinguish patients with and without PBI. By combining those two markers reduction of antibiotic misuse may be possible.

## Supplementary information

**Additional file 1: Table S1.** Sepsis markers in episodes with proven bacterial infection at admission and day 2 (*Univariate analysis*). **Table S2.** Sepsis markers in episodes without proven bacterial infection at admission and day 2 (*Univariate analysis*). **Figure S1.** Receiver operating characteristics curve of procalcitonin at day 2. **Figure S2.** Receiver operating characteristics curve of whole blood count and temperature on day 2.

## Data Availability

The datasets used and/or analyzed during the current study are available from the corresponding author on reasonable request.

## Abbreviations

AUC: Area under the curve
D0: Admission
D2: Day 2
DKA: Diabetic ketoacidosis
IC95: 95% confidence interval
ICU: Intensive care unit
IQR: Interquartile range
NLCR: Neutrophils-to-lymphocytes count ratio
PBI: Proven bacterial infection
PCT: Procalcitonin
ROC: Receiver operating characteristics
SAPS II: Simplified acute physiology score II
WBC: White blood cell count
