# Correction to: Early sepsis markers in patients admitted to intensive care unit with moderate‑to‑severe diabetic ketoacidosis

**DOI:** 10.1186/s13613-020-00691-7

**Published:** 2020-06-02

**Authors:** Florian Blanchard, Judith Charbit, Guillaume Van der Meersch, Benjamin Popoff, Adrien Picod, Regis Cohen, Frank Chemouni, Stephane Gaudry, Helene Bihan, Yves Cohen

**Affiliations:** 1 Medical-Surgical Intensive Care Unit, Avicenne University Hospital, AP-HP, Paris 13 University, Sorbonne Paris Cité, 125 rue Stalingrad, 93000 Bobigny, France; 2 Department of Endocrinology, Diabetology, Metabolic Disease, Avicenne University Hospital, AP-HP, Paris 13 University, Sorbonne Paris Cité, CRNH-IdF, 125 rue Stalingrad, Bobigny, France; 3 Department of Anesthesiology and Critical Care, Rouen University Hospital, Rouen, France; 4 Department of Endocrinology, Delafontaine Hospital, 2 rue du Dr Delafontaine, Saint-Denis, France; 5 Gustave Roussy, Médecine Intensive Réanimation, 94805 Villejuif, France; 6 Sorbonne University, INSERM, Remodeling and Repair of Renal Tissue, UMR S1155, Tenon Hospital, Paris, France

## Correction to: Ann Intensive Care (2020) 10:58 10.1186/s13613-020-00676-6

After publication of the original article [1], the authors would like to correct a percentage in the Abstract paragraph, under the heading "Results".

The sentence currently reads: “At admission, procalcitonin (median: 3.58 ng/mL vs 0.52 ng/mL, p < 0.001) and presence of fever (25% vs 44%, p = 0.007) were different between episodes with and without proven bacterial infection in both univariate and multivariate analysis”.

The sentence should read: “At admission, procalcitonin (median: 3.58 ng/mL vs 0.52 ng/mL, p < 0.001) and presence of fever (25% vs 4%, p = 0.007) were different between episodes with and without proven bacterial infection in both univariate and multivariate analysis”.

The original article [1] has been updated.
