# Supplementary material for: Early sepsis markers in patients admitted to intensive care unit with moderate-to-severe diabetic ketoacidosis
Source: Ann Intensive Care. 2020 May 19;10:58. doi: 10.1186/s13613-020-00676-6 (PMC7237630; doi:10.1186/s13613-020-00676-6)
Supplement: Supplementary file 1 — Additional file 1: Table S1. Sepsis markers in episodes with proven bacterial infection at admission and day 2 (Univariate analysis). Table S2. Sepsis markers in episodes without proven bacterial infection at admission and day 2 (Univariate analysis). Figure S1. Receiver operating characteristics curve of procalcitonin at day 2. Figure S2. Receiver operating characteristics curve of whole blood count and temperature on day 2. [file 13613_2020_676_MOESM1_ESM.docx]

**Additional File**

**To:** Early sepsis markers in patients admitted to intensive care unit with moderate to severe diabetic ketoacidosis

By Blanchard et al.

**Content**

Tables

**S1.** Sepsis markers in episodes with proven bacterial infection at admission and day 2 (*Univariate analysis*).

**S2.** Sepsis markers in episodes without proven bacterial infection at admission and day 2 (*Univariate analysis*).

Figures

**S1.** Receiver operating characteristics curve of procalcitonin at day 2.

**S2.** Receiver operating characteristics curve of whole blood count and temperature on day 2.

**Table S1: Sepsis markers in episodes with proven bacterial infection at admission and day 2 (*Univariate analysis*).**

| Variables | Admission | Day 2 | p-value* |
| --- | --- | --- | --- |
| Temperature, °C, median [IQR] | 36.9 [36.2-38.0] | 38.4 [37.1-39.0] | **0.019** |
| Fever^‡^, n (%) | 5 (25%) | 11 (60%) | **0.046** |
| Hypothermia^§^, n (%) | 4 (20%) | 0 (0%) |  |
| WBC, G/L, median [IQR] | 16.85 [14.25-22.15] | 13.05 [8.68-18.23] | **0.008** |
| Leukocyte abnormalities^$^, n (%) | 18 (90%) | 11 (55%) | 0.070 |
| Neutrophils count, G/L, median [IQR] | 13.30 [12.01-18.24] | 10.79 [7.39-16.64] | **0.009** |
| NLCR; median [IQR] | 14.04 [8.79-19.07] | 11.54 [7.63-23.99] | 0.985 |
| Procalcitonin, ng/mL, median [IQR] | 3.58 [1.87-11.24] | 7.43 [2.63-22.70] | 0.860 |

*****Significant difference (p<0.05) between admission and day 2 are reported in the “p-value” column. **^‡^**Fever: Temperature > 38°C. **^§^**Hypothermia: Temperature < 36°C. ^$^Leukocyte abnormalities: white blood cell count > 12000/mm3 or < 4000/mm3. IQR: interquartile range 25-75%, WBC: white blood cell count, NLCR: neutrophils-to-lymphocytes count ratio.

**Table S2: Sepsis markers in episodes without proven bacterial infection at admission and day 2 (*Univariate analysis*).**

| Variables | Admission | Day 2 | p-value* |
| --- | --- | --- | --- |
| Temperature, °C, median [IQR] | 36.4 [35.7-36.8] | 37.0 [36.8-37.3] | **<0.001** |
| Fever^‡^, n (%) | 3 (4%) | 7 (9%) | 0.131 |
| Hypothermia^§^, n (%) | 26 (32%) | 1 (1%) | **<0.001** |
| WBC, G/L, median [IQR] | 15.40 [12.30-22.50] | 8.15 [6.68-10.20] | **<0.001** |
| Leukocyte abnormalities^$^, n (%) | 62 (76%) | 14 (17%) | **<0.001** |
| Neutrophils count, G/L, median [IQR] | 13.71 [9.69-20.88] | 5.38 [3.60-7.62] | **<0.001** |
| NLCR; median [IQR] | 11.40 [5.78-19.27] | 2.84 [1.56-4.96] | **<0.001** |
| Procalcitonin, ng/mL, median [IQR] | 0.52 [0.19-1.38] | 0.42 [0.14-1.42] | **0.014** |

*****Significant difference (p<0.05) between admission and day 2 are reported in the “p-value” column. **^‡^**Fever: Temperature > 38°C. **^§^**Hypothermia: Temperature < 36°C. ^$^Leukocyte abnormalities: white blood cell count > 12000/mm3 or < 4000/mm3. IQR: interquartile range 25-75%, WBC: white blood cell count, NLCR: neutrophils-to-lymphocytes count ratio.


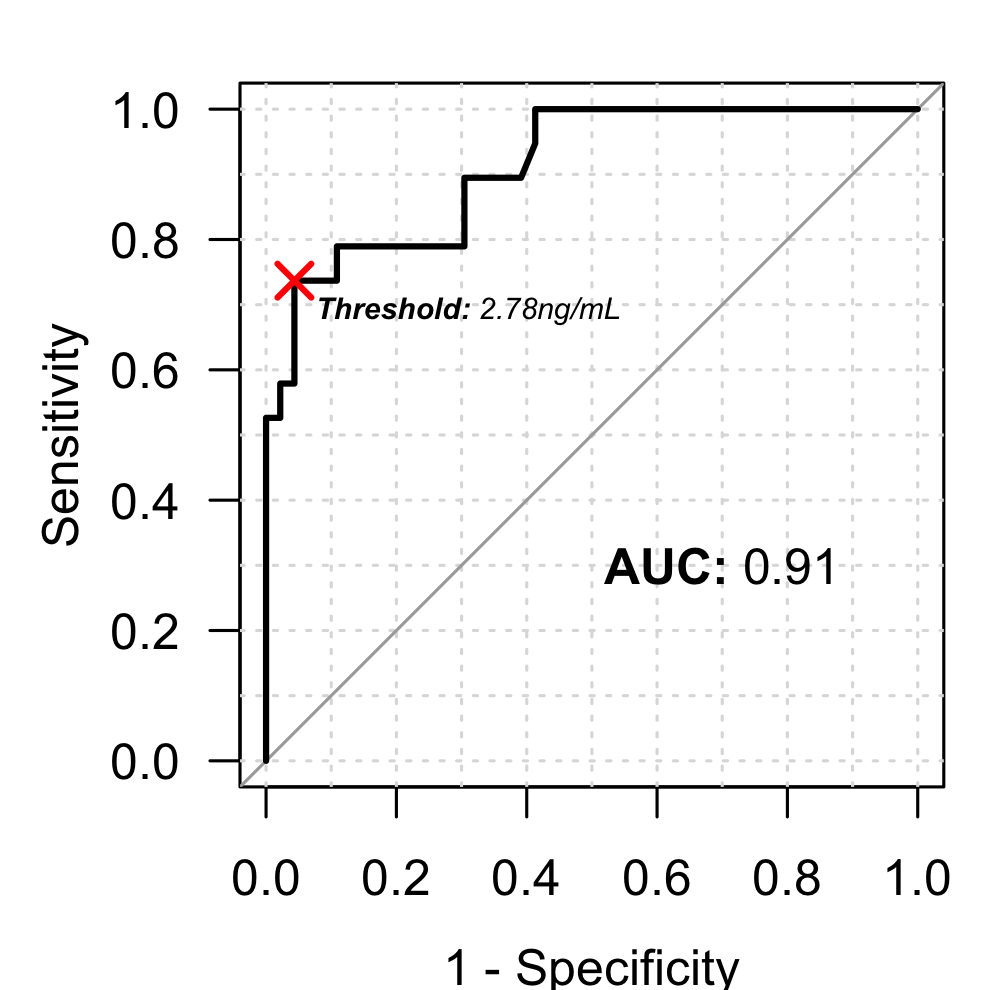


**Figure S1:** **Receiver operating characteristics curve of procalcitonin on day 2.** The area under curve (AUC) was 0.91 with optimal cutoff at 2.78ng/mL leading to a sensibility and specificity of 0.74 (IC95 [0.53-0.89]) and 0.96 (IC95 [0.89-1.00]) respectively. Positive and negative likelihood ratio were 18.5 and 0.25 respectively.

**Figure S2:** **Receiver operating characteristics curve of whole blood count and temperature on day 2.**
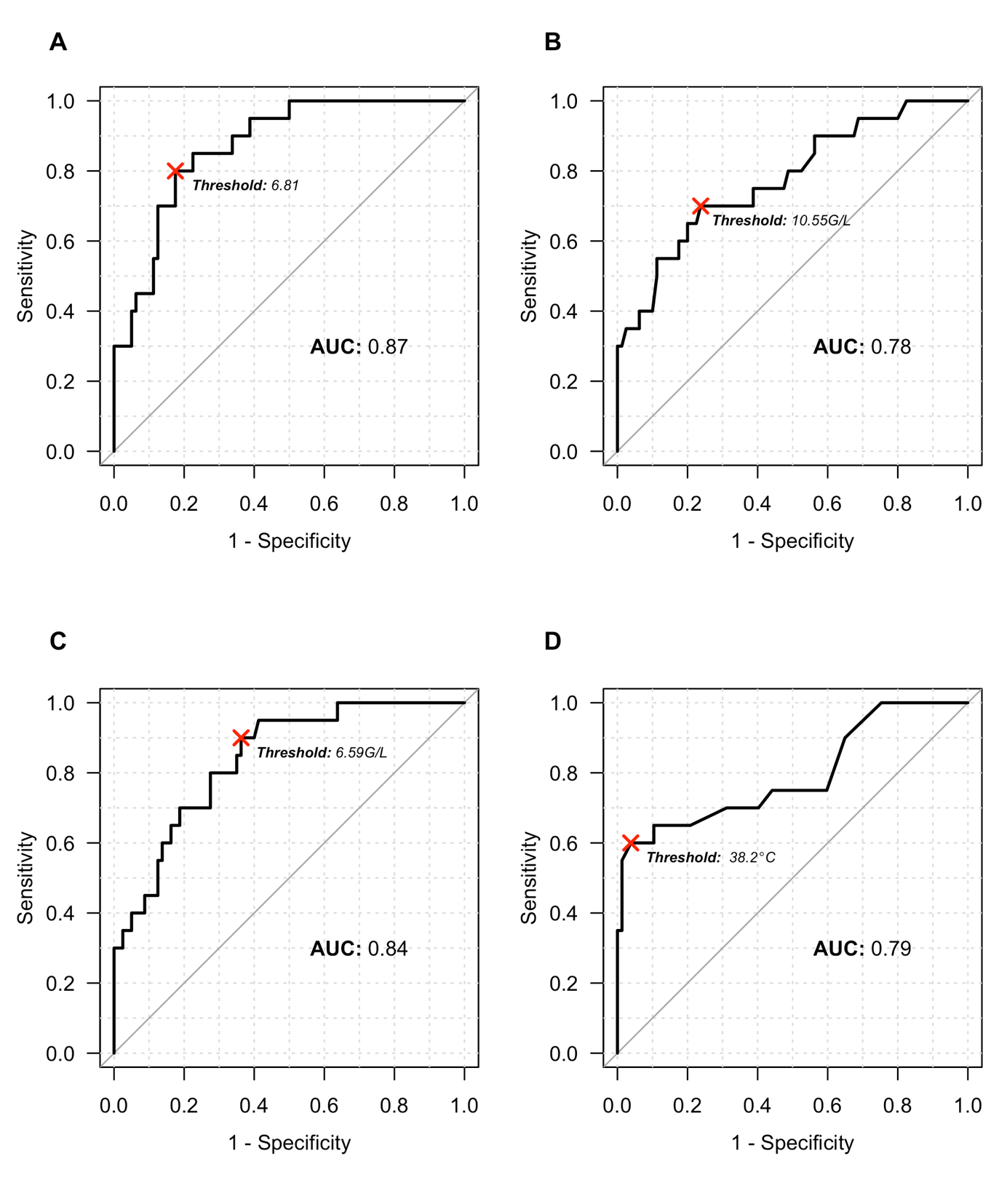
 Receiver operating characteristics curve of neutrophils-to-lymphocytes count ratio, white blood cell count, neutrophil count and temperature are represented in (A), (B), (C) and (D) respectively. (A) The area under curve (AUC) of the *neutrophils-to-lymphocytes count ratio* was 0.87 (IC95 [0.80-0.95]) with optimal cutoff at 6.81 leading to a sensibility (Se) and specificity (Sp) of 0.80 (IC95 [0.60-0.95]) and 0.83 (IC95 [0.74-0.83]). (B) The AUC of the *white blood cell count* was 0.78 (IC95 [0.65-0.90]) with optimal cutoff at 10.55G/L leading to a Se and Sp of 0.70 (IC95 [0.50-0.90]) and 0.76 (IC95 [0.68-0.85]). (C) The AUC of the *neutrophil count* was 0.84 (IC95 [0.75-0.93]) with optimal cutoff at 6.59G/L leading to a Se and Sp of 0.90 (IC95 [0.75-1.00]) and 0.64 (IC95 [0.53-0.74]). (D) The AUC of the *temperature* was 0.79 with optimal cutoff at 38.2ºC leading to a Se and Sp of 0.60 (IC95 [0.40-0.80]) and 0.96 (IC95 [0.92-1.00]).
